# Supporting implementation science capacity in a university setting

**DOI:** 10.1186/1748-5908-10-S1-A79

**Published:** 2015-08-20

**Authors:** Rinad Beidas, Ian Bennett, Linda Fleischer, Theodore Day, Zachary Meisel

**Affiliations:** 1 University of Pennsylvania, Philadelphia, PA, 19104, USA; 2 Children's Hospital of Philadelphia, Philadelphia, PA, 19104, USA

## 

While dissemination and implementation (DI) science has become a clear area of research focus with associated methodologies, grant programs, journals, and training programs, these are relatively recent developments which have created a gap in training and career development. Many large institutions with major research programs have some investigators who are carrying out DI research but these tend to be limited and many investigators whose work has relevance to the field are not trained adequately to take advantage of existing grant mechanisms. The University of Pennsylvania has taken the strategy of creating the Implementation Science Working Group (ISWG) with the goal of providing an opportunity for idea development, feedback, grant review, and review of key methodologic approaches and conceptual frameworks for the field. A key element of the ISWG has been to support pilot studies by faculty from across the university which focus on DI topics and which have potential for translation into NIH grant applications. In this presentation, we discuss current efforts of the Penn ISWG as an exemplar for other institutions, and present examples of pilot grant receipts who have received funding to conduct DI related work within the context of the university efforts to support DI capacity.

